# Changes in Biomass and Quality of Alpine Steppe in Response to N & P Fertilization in the Tibetan Plateau

**DOI:** 10.1371/journal.pone.0156146

**Published:** 2016-05-25

**Authors:** Junfu Dong, Xiaoyong Cui, Shuping Wang, Fang Wang, Zhe Pang, Ning Xu, Guoqiang Zhao, Shiping Wang

**Affiliations:** 1 College of Resources and Environment, University of Chinese Academy of Sciences, Beijing 100049, China; 2 College of Life Sciences, University of Chinese Academy of Sciences, Beijing 100049, China; 3 Institute of Tibetan Plateau Research, Chinese Academy of Sciences, Beijing 100101, China; Institute of Tibetan Plateau Research, CHINA

## Abstract

In the alpine steppe zone on the Central Tibetan Plateau, a large amount of area has been degraded due to natural and artificial factors. N & P fertilization is widely accepted to recover degraded pastures in other regions all over the world. However, it is not clear how alpine steppe communities respond to N & P fertilization, and what is the optimal application rate, in the perspective of forage production. To attempt to explore these questions, in July 2013, two fencing sites were designed in Baingoin County with 12 treatments of different levels of nitrogen (N_0_: 0; N_1_: 7.5 g m^-2^ yr^-1^; N_2_: 15 g m^-2^ yr^-1^) & phosphate (P_0_: 0; P_1_: 7.5 gP_2_O_5_ m^-2^ yr^-1^; P_2_: 15 gP_2_O_5_ m^-2^ yr^-1^; P_3_: 30 gP_2_O_5_ m^-2^ yr^-1^). The results indicated N&P addition was capable to ameliorate the quality of the two sites in the Tibetan Plateau steppe. Increasing N application level resulted in significant increment in Gramineae and total biomass in the two sites. P addition significantly improved the quantity of Compositae, total biomass and the biomasss of other species in site II, while it only significantly improved the total biomass in site I. Gramineae was much more sensitive to N-induced changes than P-induced changes, and this indicated N addition was better to ameliorate the quality of plateau steppe than P-induced changes. No strong evidence was found for critical threshold within 15 g N m^-2^ yr^-1^, and there was decreasing tendency when P addition rate was above 15 g m^-2^ yr^-1^. N&P has the potential to accelerate soil acidification, which improved the content of available K, likely as a result of nonsignificant correlation between biomass and soil moisture. This work highlights the the tradeoffs that exist in N and P addition in recovering degraded steppe.

## Introduction

Recently, with the development of urbanization and intensified grazing, degradation has become more and more severe in the Tibetan Plateau [1]. Despite being as “the third pole of the earth” (average elevation 4000 m a.s.l.) [2] and the youngest plateau in the world, which extends over 2.5 million km^2^ (almost a quarter of the size of China or the United States of America) [3–5], the plateau receives less attention than their counterparts in the boreal and tropical regions [6]. Though the world’s highest grassland was found in the Chang Tang Plateau (including Nagchu and Ali prefectures with an average elevation of over 4500 m, and it covers approximately 600 000 km^2^) on the Tibetan plateau [7], most previous studies on these places were focused on the plateau meadow. 77.1% of the total area degraded in this region due to overgrazing [8]. Recovering these degraded grassland is extremely urgent, because of its important ecological values and economic values.

Degradation has reduced the proportion of forage grasses [9], contributed to the ssssdecline of biomass [10,11], and decreased the proportion of reproductive females [11]. One of the most important factor of degradation is overgrazing. In the alpine meadow, some studies have found that long-term grazing could cause an increase in pH [12], which considerably effect the availability of plant nutrients. Soil organic matter would also decrease by 0–10 cm with the increase of grazing [13].With the increase of the population and livestock, it is infeasible to prohibit grazing of the entire plateau. In the Tibetan Plateau, low levels of N and P may have limited the increase of aboveground biomass [14]. According to Liebig’s law of the minimum, the scarcest resource that the plant needs is its most limiting factor [15]. Fertilization can provide the necessary nutrients for plants and increase the grass yield [16,17]. Nitrogen and phosphorus are common essential elements and usually constrain plant productivity in most terrestrial ecosystems [18]. And the addition of N may exacerbate P limitation to plant growth [19]. While most studies have been conducted on alpine meadow [20,21], little is known about the interactive effect of N and P on alpine steppe [22–24]. Until now, the deposition of reactive N has doubled over the last century, and it is projected that N deposition would increase another two- or threefold in the coming decades [18,25].

Currently, N & P fertilization is widely accepted to recover degraded pastures in other regions all over the world [26–29]. Some studies have found that with adding nitrogen and phosphorus can increase shoot biomass and decrease the ration of roots after observing shoot biomass after 8 years [21]. Studies also reported that nitrogen and phosphorus fertilization could increase biomass significantly and increase the proportion of Gramineae [16,30] [17], although there are notable exceptions showed that increased N does not always result in an increment of aboveground biomass [31]. These differences may correlated with various plant species [32] and ecosystem types.

In this study, we focus on two typical steppe, one is dominated by *Stipa purpurea* and the other is dormnated by *L*. *leontopodioide*. We aim to answer the following questions: 1) How do alpine steppe communities respond to N & P fertilization? 2) What dosage is effective and economical, in the perspective of forage production? 3) Whether there exist any difference between the two typical steppes after N & P fertilizer addition? 4) And whether there are interaction between N and P fertilization? The findings of this study will shed light on the efficiency of fertilization in the recovery of the Tibetan Plateau steppe.

## Material and Methods

### Study Site

The field experiment was conducted in alpine steppe at a mean altitude of 4678 m above sea level, which is located in Baingoin County (N31°26’, E90°02’) in northern Tibet. The location is semiarid cold alpine steppe, and the soil is alpine steppe soil [33]. According to the local observatory (31°22′N, 90°01′E, 4700 m), the annual sunshine duration is 3210.3 h, the mean annual temperature is -1.2°C with mean monthly temperatures ranging from -17.5°C in January to 14.7°C in July. The annual precipitation ranges from 289 to 390 mm and has a mean value of 301.2 mm, which falls by 80% from June to September. The annual evaporation is 1993.4 to 2104.3 mm.

On a 5.5° gentle slope, two sites were chosen. No fertilizer had been applied in either site before this study. Site I is dominated by the species of *Stipa purpurea*, accompanied with *Leontopodium leontopodioide* and *Heteropappus bowerii*. In site II, the dominant species is *L*. *leontopodioide*, accompanied with *H*. *bowerii* and *S*. *purpurea*. Site I and II were fenced with an area of 100 ×100 m to exclude large animals in July 2013.

### Experimental Design

Identical treatments were applied to site I and site II. At each site, 60 5×5 m plots were laid out in a randomized design, and plots were separated by 2-meter buffer zones. To avoid edge effects, each plot was placed at least 3 m inside each site. There were 5 replicates for each of the 12 treatments which included 3 levels of N (N_0_: 0; N_1_: 7.5 gN.m^-2^.yr^-1^; N_2_: 15 gN.m^-2^.yr^-1^) and 4 levels of P (P_0_: 0; P_1_: 7.5 gP_2_O_5_.m^-2^.yr^-1^; P_2_: 15 gP_2_O_5_.m^-2^.yr^-1^; P_3_: 30 gP_2_O_5_.m^-2^.yr^-1^) as commercial fertilizers of urea and triple super-phosphate. Fertilizers were applied evenly at different times: 1) initial growth season in July; 2) vigorous growth season in August.

### Field sampling and measurements

In accordance with earlier studies, the total species were divided into 5 communities: Gramineae (including *S*. *purpurea*, *Poaannual*, *Festuca coelestis*), Compositae (including *L*. *leontopodioide*, *Heteropappus Puppyflower*), Cyperaceae (including *C*.*oxyleuca V*.*Krecz*, *Carex moorcroftii*, *Kobresia pygmaea*), Rosaceae (including *Potentilla bifurca Linn*., *Potentilla multifida*) and forbs (including *Sickle pod beans jujube*, *Rhodiola rosea L*., *Androsace mariae* and so on). Since our preliminary analysis found no significant effects of N&P addition existed on the responses of Cyperaceae, Rosaceae and forbs, so we classified them together as other species in this study.

Aboveground vegetation was sampled twice in July and September in 2014 by clipping all plants at the soil surface of each plot. To minimize the disturbance to vegetation and soil, we used 1×1 m square sampling plots to investigate the aboveground biomass repetitions [34]. All of the plants samples were sorted to species, de-enzymed at 85°C for 30 min and oven-dried at 65°C until a constant weight was achieved. Afterwards, the samples were weighed on an electronic scale (accurate to one hundredth of a gram). The aboveground biomass was determined by adding the dry weight of each community in every plot.

Soil samples were taken from the surface to a depth of 10 cm in early September, and were stored in a refrigerator at 4°C. For each site, seven soil cores were collected using a 3-cm diameter soil auger and mixed in situ into one composite sample.

Soil pH was measured with a soil to water ratio of 1:2.5; soil organic matter was determined by potassium dichromate oxidation [35]; total nitrogen (N) was determined by the Kjeldahl method [36]; and soil available nitrogen, total phosphorus and available phosphorus were measured by the methods of Miller and Keeney [37]. Within 10 days of the colletion, the soil samples were extrated with a 2 M KCl solution, and ammonium (NH_4_^+^) concentration was measured by colorimetry on a SMARTCHEM 140 (Italy). Concentration of extractable soil NH_4_^+^-N was expressed as milligrams per kilogram on the basis of dry soil mass. Soil moisture was determined using the gravimetric method. The soil samples were weighed before and after being oven-dried at 105°C for 48 h. All the properties of soil before fertolized are as below (**Table 1**).

**Table 1:** Soil properties (means ±SEM) of site I and site II before treatment.

	Term	Site Ⅰ	Site Ⅱ
Soil property (0–10 cm)	TC(g/Kg)	32.53±0.56	31.67±0.37
	TN(g/Kg)	1.65±0.09	7.51±0.00
	TP(g/Kg)	0.62±001	1.71±0.00
	AN(mg/Kg)	128.17±5.11	0.50±0.00
	AP(mg/Kg)	4.96±0.25	131.27±8.25
	pH	6.97±.01	4.55±0.18
Soil property (10–20 cm)	TC(g/Kg)	18.80±0.69	26.11±0.23
	TN(g/Kg)	1.09±0.10	7.27±0.01
	TP(g/Kg)	0.74±0.01	1.10±0.10
	AN(mg/Kg)	77.30±2.60	045±0.16
	AP(mg/Kg)	3.04±0.14	95.81±1.42
	pH	7.04±0.01	4.55±0.14

Note: TC = soil organic matter, TN = total nitrogen, TP = total phosphorus, AN = available nitrogen, AP = available phosphorus.

### Statistical Analysis

All statistical analyses were performed using SPSS version 16.0 (SPSS Inc., Chicago, IL, USA) and Origin 8.0 (Origin Lab Corporation, USA). ANCOVAs were performed to examine the significance of factors’ effects and their interactions on the observed parameters. Duncan’s protected least significant difference test was applied to examine the quantitative differences between treatments. Analyses across sites were performed using General Linear Model for N addition rate, P addition rate and their interaction as fixed-effects. After that, no significant difference were found between the biomass among the 12 treatments in July, while the interaction of N addition and sampling sites had significant influence on biomass. Thus, we ran additional analyses by ANOVA of the biomass in September for each site to determine the response of pattern and magnitude. Regression models with N or P as a continuous variable were used to determine the general relationship between N or P addition and various response (i.e. to estimate threshold levels). Those ANOVAS were followed by a Duncan’s multiple-ranges test to compare the N-addition or P-addition effects for each rate.

## Results

### Aboveground Biomass of Different N&P Addition Rate

ANCOVAS of aboveground biomass, using sites, N addition, P addition and all their interactions as fixed factors, and biomass in July as covariate, showed that the effects of different sites was highly significant on biomass of Compositae, other species and total biomass (Table 2). The average aboveground biomass of Compositae, other species and total biomass in site II was 171.2%, 248.0% and 103.4% higher compared to that in site I.

**Table 2:** The significant of different sites, N and P to functional groups.

Response	Term	Df	F	P
Gramineae	Biomass in July	1	.070	.792
	Site (S)	1	.451	.504
	N–treatment (N)	2	9.255	< 0.01
	P-treatent (P)	3	1.843	.147
	S×N	2	.088	.916
	S×P	3	.519	.670
	N×P	6	.577	.747
	S×N×P	6	.134	.991
Compositae	Biomass in July	1	2.107	.151
	Site (S)	1	17.465	< 0.01
	N–treatment (N)	2	5.852	< 0.01
	P-treatent (P)	3	3.980	< 0.05
	S×N	2	3.862	< 0.05
	S×P	3	.366	.778
	N×P	6	.706	.646
	S×N×P	6	.593	.735
Other	Biomass in July	1	.354	.554
	Site (S)	1	26.706	< 0.01
	N–treatment (N)	2	1.316	.275
	P-treatent (P)	3	2.110	.107
	S×N	2	.954	.390
	S×P	3	.725	.541
	N×P	6	1.244	.295
	S×N×P	6	.934	.476
Total	Biomass in July	1	3.952	.051
	Site (S)	1	85.557	< 0.01
	N–treatment (N)	2	20.750	< 0.01
	P-treatent (P)	3	9.967	< 0.01
	S×N	2	4.039	.022
	S×P	3	1.535	.213
	N×P	6	1.723	.128
	S×N×P	6	.759	.604

Note: There are 71 degrees of freedom for error.

**Fig 1:**
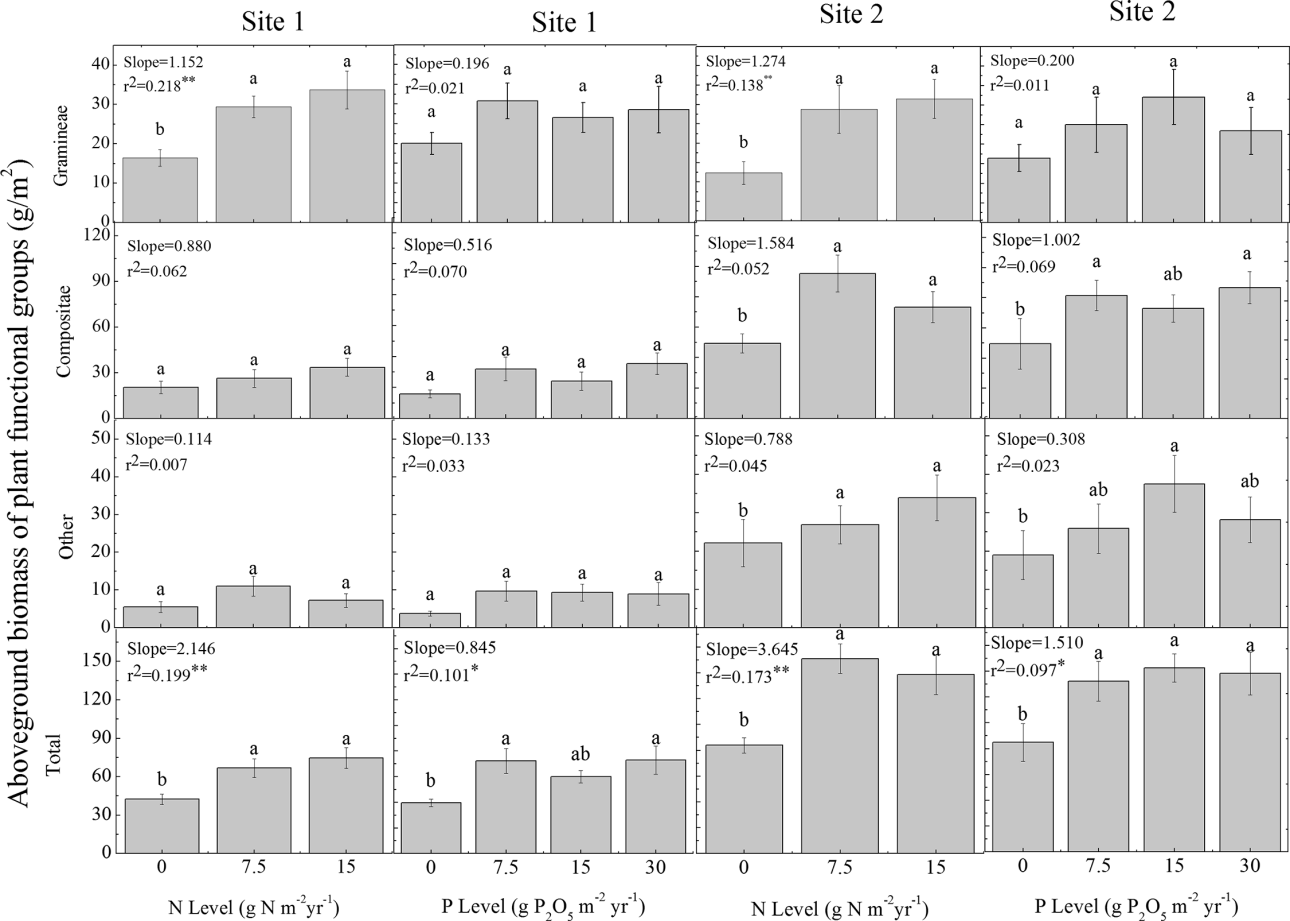
Difference of N&P rate on aboveground biomass of plant functional groups (error bars denote SEM). Aboveground biomass for each plant functional group of each addition rate was the average of sixteen replicates of the four treatments for N addition, and twelve replicates of three treatments for P addition. Bars with the same letter were not significantly different in Duncan’s multiple range tests reported from ANOVA (*P* > 0.05). For both site, regression parameters were estimated aboveground biomass using linear model with N or P treatment as a continuous preditor, i.e. Aboveground biomass = Intercept + Slope ×addition rate (N or P). Significant differences are reported as ^*^, *P* < 0.05; ^**^, *P* < 0.01.

Aboveground biomass tended to increase significantly at all N addition rate in site II, while significant increase only exist in Gramineae and total biomass in site I. However, no significant increase was found in various species with different levels of P addition whereas the total biomass increase significantly (Fig 1).

Site II (Gramineae biomass accounted for 19.33% of the total biomass with a range from 7.25% to 34.31%; Compositae biomass accounted for 58.42% of the total biomass with a range from 43.18% to 79.19%; biomass of other species accounted for 22.25% of the total biomass with a range from13.56% to 42.53%), dominant species is *L*. *leontopodioide*, has higher aboveground biomass of plant functional groups than site I except Gramineae (9.41% lower than site I). The interactions between N, P and different sites were nonsignificant except the interaction between different sites and N addition rate for Compositae (Table 2). In site II, aboveground biomass of all plant functional groups showed significant difference between control and N addition. And there were linear correlation between N addition and biomass for Gramineae and total significantly. And the rate of P application only had linear correlation with total biomass significantly in site II (Fig 1).

In site I (Gramineae biomass accounted for 43.63% of the total biomass with a range from 29.09% to 56.30%; Compositae biomass accounted for 43.53% of the total biomass with a range from 40.22% to 57.5%; biomass of other species accounted for 12.84% of the total biomass with a range from5.24% to 24.46%), with the increasing of N addition rate, the biomass of Gramineae and total biomass showed highly significant increase (Gramineae: t^2^ = 0.218; total biomass: t^2^ = 0.199). For different rate of P addition, only total biomass showed significant linear correlation with P rate (r^2^ = 0.101, significant) (Fig 1).

Our experiment in the Tibetan Plateau demonstrated that the qualitative effects of N&P addition were similar between the two sites, whereas the quantitative effects in site II was more significant. When added N rate till 7.5 g m^-2^ yr^-1^, the effects showed significant compared to the control, and when added N rate at 15 g m^-2^ yr^-1^, the effects were same to the rate of 7.5 g m^-2^ yr^-1^. There was nonsignificant effects for P addition in site I for most plant except the total biomass, while the effects of P addition in site II was more significant, and the threshold was 7.5 g m^-2^ yr^-1^ for Compositae and total biomass, 15 g m^-2^ yr^-1^ for other species (the total biomass except Compositae and Gramineae). Thus, throughout the study period, the Site II showed a more sensitiveresponse than site I.

### Aboveground Biomass of Different Treatment

**Fig 2:**
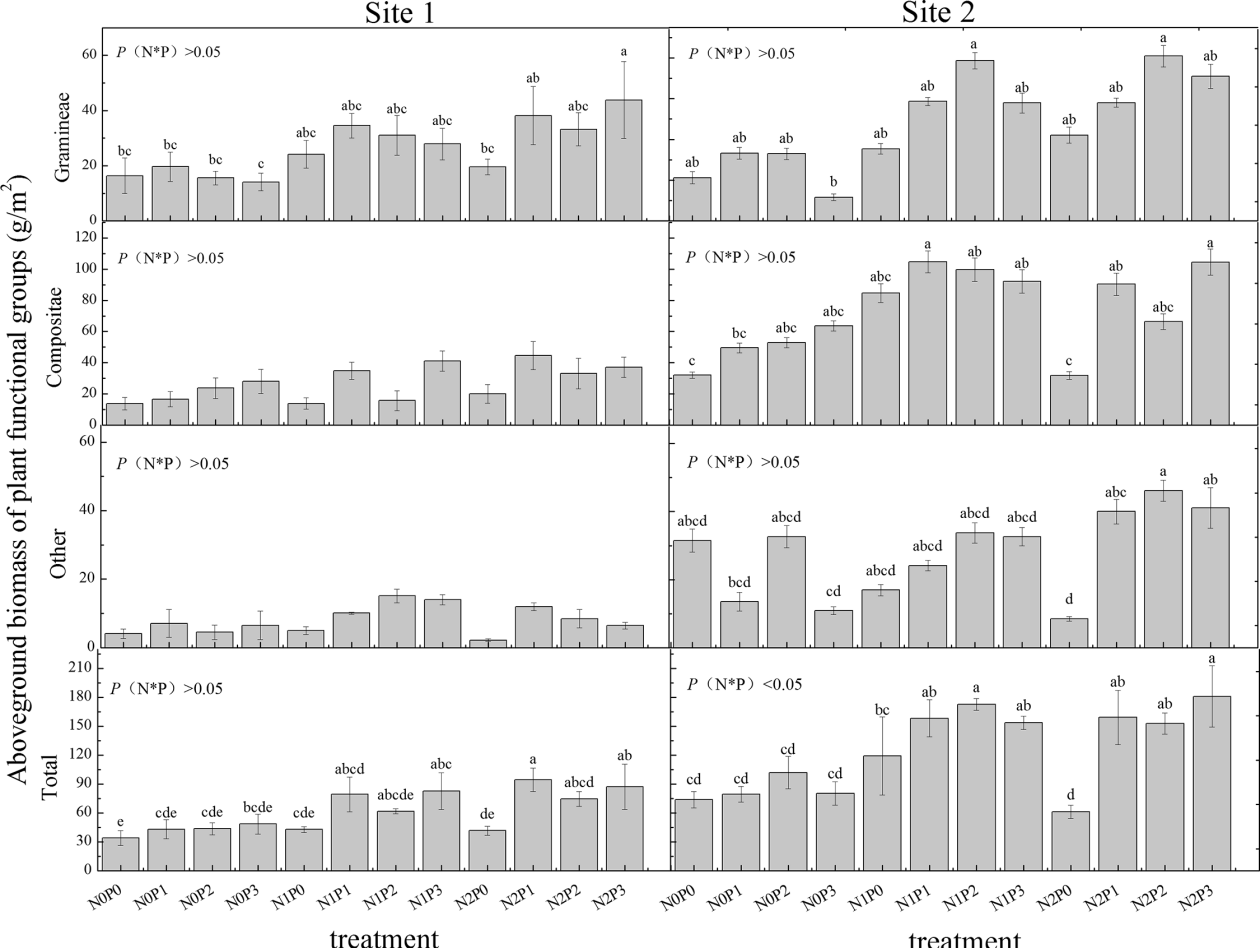
Difference of different treatment on aboveground biomass of plant functional groups. Aboveground biomass for each treatment was the average of four replicates (error bars denote SEM), and *P* (N*P) indicates the interaction between N and P addition.

Acordinng to the above results, we took further analyses for different treatment in site I and site II. For all the plant functional groups in the two sites, N or P added alone at higher rate hada decreasing tendency compared to the control (i.e. decrease: Gramineae in the two sites and other species in site I when P addition rate was 30 g m^-2^ yr^-1^, and other species in two sites and the total biomass in site II when N addition rate was 15 g m^-2^ yr^-1^). Except the above treatment, the other treatment of N or P added alone also had nonsignificant effect compared to the control (Fig 2).

Our experiment showed that the combined addition of N and P was better than added alone, eventhough their quantitative effects varied substantially among different treatment.

### The Ratio of Gramineae to Compositae

**Fig 3:**
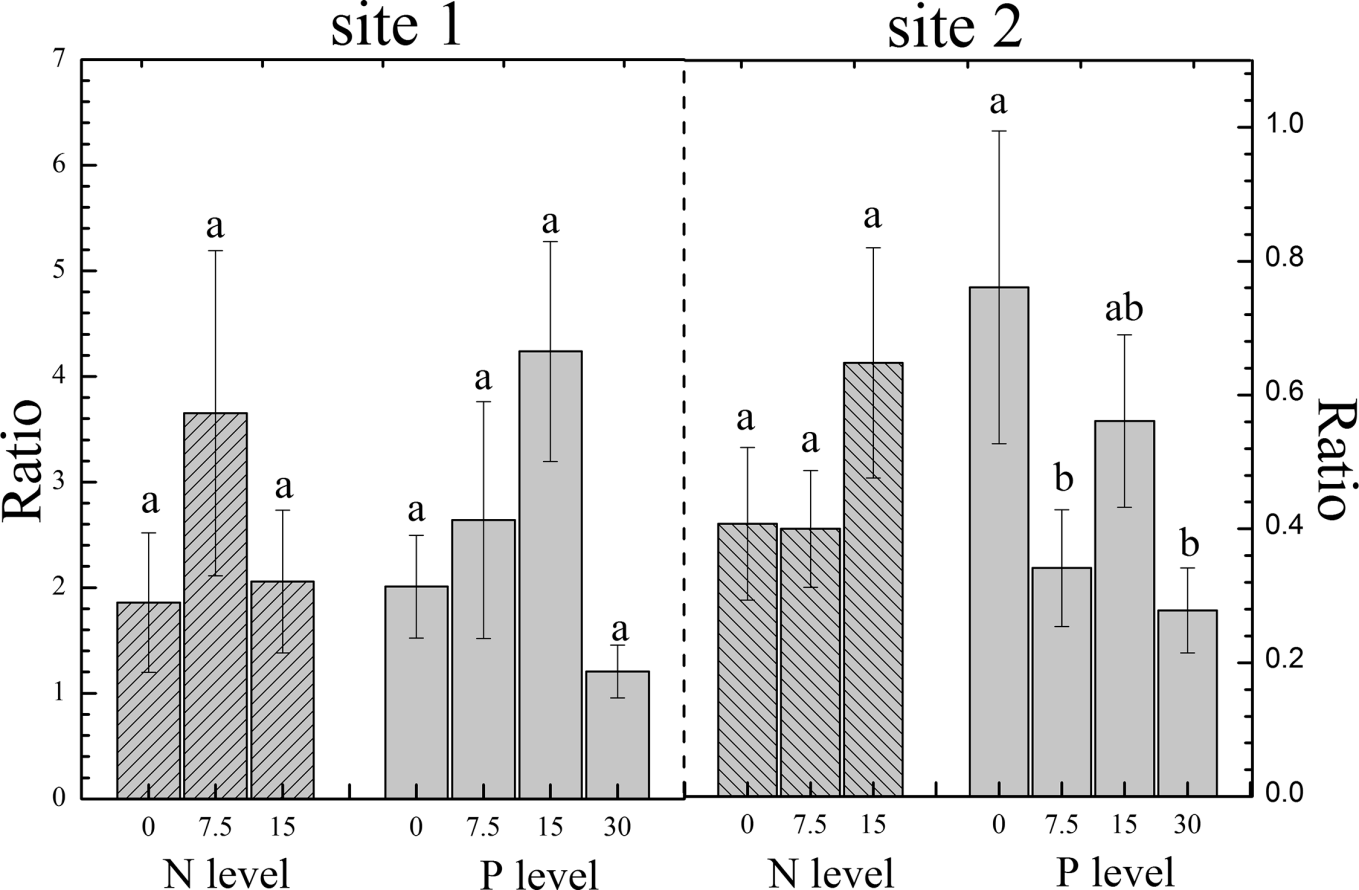
Ratio of Gramineae to Compositae (error bars denote SEM). Medium pattern with grey back indicates the ratio of different N level, and the only grey back indicates the ratio of different P level.

The ratio of Gramineae to Comositae could represent the quality of grassland at some aspect, since livestocks (i.e. sheep and yak) like to feed on Gramineaerather than Compositae. In site I wherethe biomass of Gramineae was more than Compositae, we didn’t find any significant difference between different N or P level, while wefound there was a higher ratio when N addition rate was 7.5 g m-2 yr-1 and P addition rate was 7.5 or 15 g m-2 yr-1 compared to the control (Fig 3).

In site II where the biomass ofGramineae was less than Compositae, our experiment results showed that the increament of P addition rate could decrease the relative quality in the total biomass significantly. And the N level of 15 g m-2 yr-1 could increase the ratio of Gramineae, though it was nonsignificant (Fig 3).

### The Physical and Chemical Properties of Soil

**Fig 4:**
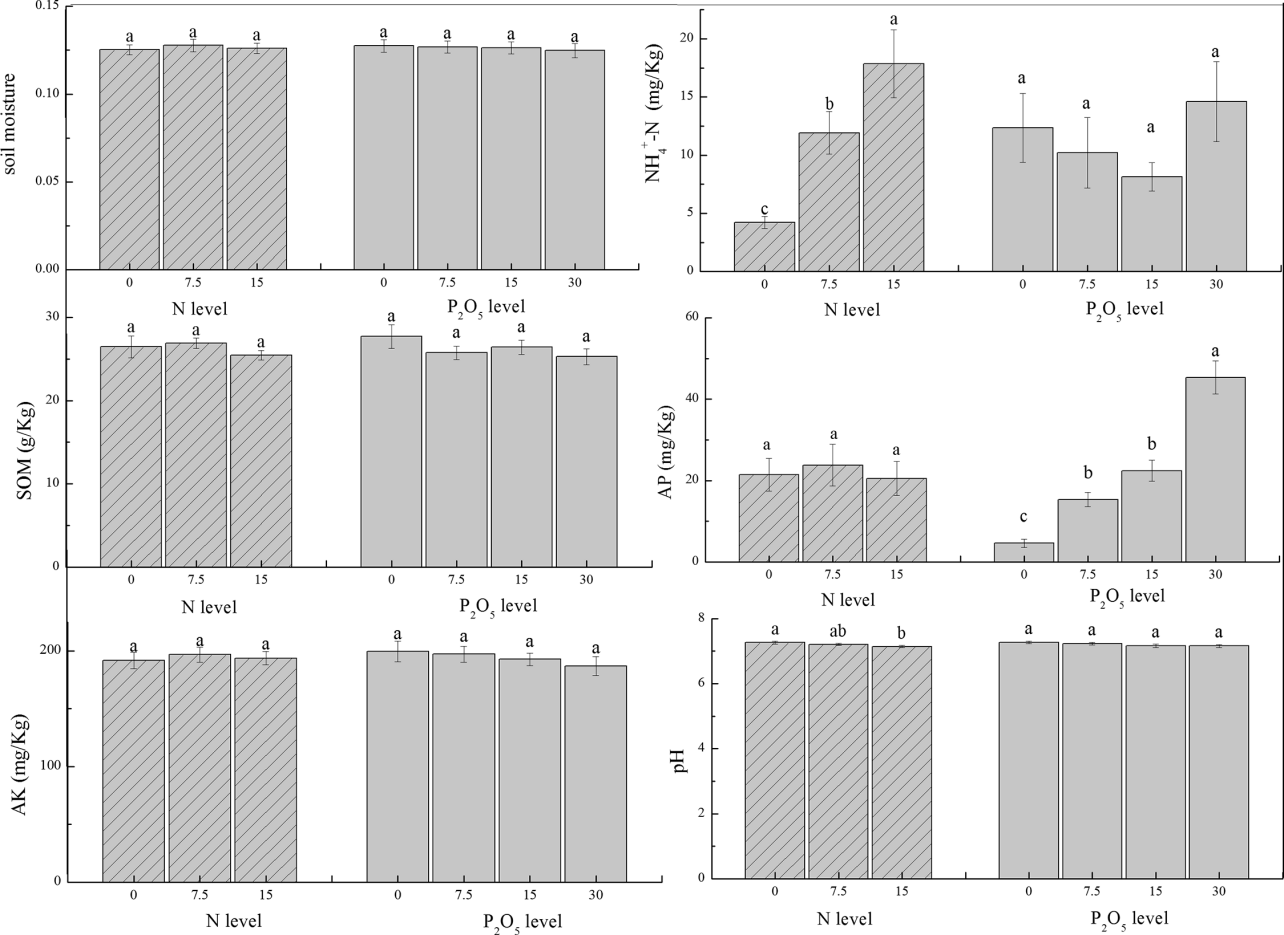
The physical and chemical properties of soil in site I (error bars indicate SEM). SOM as soil organic matter, AP as available phosphorus, AK as available kalium.

The quality of the steppe in site I was better than site II, beause there was more Gramineae. We had further analysis for the physical and chemical properities of the soil (0–10 cm) for site I. Our experiment showed that there were nonsignificant differences in the interaction of N and P for the soil physical and chemical properties based on our analyses. Soil NH_4_^+^-N tended to increase significangtly at all rates of N addition compared to the control. Soil AP also had a highly significance at different P application levels compared to control, while P_3_ has significantly difference with P_1_ and P_2_. Our results also indicated that there was a decreasing tendency for pH with the increasing of N addition rate (Fig 4).

We applied further analysis of multiple linear regression (MLR) between TB (total biomass), GB (Gramineae biomass), CB (Compositae biomass) and different properities of soil. Three MLR models were developed for the predication of TB, GB and CB, respectively (Table 3).

**Table 3:** Multiple linear regression between TB and properities of soil.

	GB	CB	TB	moi	NH4+-N	SOM	AP	AK
moi	.045	-.119	-.069					
NH4+-N	.454**	.174	.254*	.051				
SOM	-.157	.011	-.079	.477**	-.097			
AP	.156	.353**	.430**	.089	.149	-.073		
AK	.048	-.047	-.115	.331*	.037	.415*	.037	
pH	-.074	-.229	-.244*	-.317*	-.254*	-.108	-.364**	-.179

*P < 0.05

**P < 0.01, GB as Gramineae’s biomass, CB as Compositae’s biomass, TB as total biomass, moi as the moisture of soil, SOM as soil organic matter, AP as the available phosphorus in soil, and AK as the available kalium in soil.

The models obtained were the following:

$$TB=44.285+0.720\times c(\text{AP})\left({\text{R}}^{2}=0.185,\;\text{t}=3.199,P=0.003\right)$$

$$GB=18.393+0.716\times c({NH}_{4}^{\;+}-N)\left({\text{R}}^{2}=0.206,\;\text{t}=3.455,P=0.001\right)$$

$$CB=17..113+0.442\times c(AP)\left({\text{R}}^{2}=0.124,\;\text{t}=2.557,P=0.014\right)$$

For these models, only the explanatory variables which presented parameters with statistical significance were considered. Forthe total biomass, the AP content showed significant influence, while other properities had nonsignificant influence on the total biomass. The content of NH_4_^+^-N had a significant influence on the biomass of Gramineae. The same to total biomass, content of AP had a sigificant influence on the biomass of Compositae.

For the other soil properities, we could conclude that there were highly significant positive correlation between SOM and moisture, while there was a negative correlation between pH and AP. There were significant correlation for moisture with AK and pH. There was negative correlation between content of NH_4_^+^-N and pH. We could also conclude that there was a higher content of AK with the increase of SOM.

## Discussion

In spite of numerous efforts that have been undertaken to arrest land desertification in China, grassland degradation is advancing over wide areas through overgrazing, climate change, cropland misuse and unregulated collection of fuel and medical plants [38].

Our results showed that the N or P addition could significantly increase the total biomass of the Tibetan Plateau steppe, while there were different responses among various plant functional groups. In site I and site II, any application rates of N showed significantly increase of the biomass of Gramineae, while no significant increase were found with P addition. Compositae showed nonsignificant difference at any addition rate of N or P in site I, while it was significant difference in site II. The other species also has the same tendency with Compositae. We can conclude that Gramineae tend to be more sensitive to N addition than P addition in spite of the dominant species. This finding can also be demonstrated by the MLR’s results. Compositae only showed significant difference in site II where it is dominated. The other species showed significant difference in site II, while it was nonsignificantin site I. These patterns suggest five conclusions. First, N addition could significantly increase the biomass of Gramineae, which could improve the Tibetan Plateau steppe’s quality.However, we didn’t find strong evidence for critical threshold within the range tested here for Gramineae, maybe N addition rate was below this threshold and Gramineae need more nitrogen input, orhis may due to microbial and abiotic processes that outcompeted plants for excess N[39]. Second, the Compostae could not show significant difference if it was not the dominate plant functional group or the biomass was lower than 43.53%. And the higher level of N or P could decrease the biomass. Third, other functional groups showed significant influence if its biomass overpassing 22.25% of the total biomass. Forth, application of N (15 g m^-2^ yr^-1^) or P (30 g m^-2^ yr^-1^) alone had toxic effects. Fifth, the difference observed between the two sites suggest that site-species dynamics modulate the impact of input of N or P [39].

Fertilization experiments provide effective ways of examining the nutritional status of ecosystems and have been conducted to test the effects of N addition on biomass [39]. Our results showed that N and P, which are limited in the alpine meadow [30], are also limited elements in the Tibetan alpine steppe. Some studies have shown that there are lower N/P and P levels compared to the whole terrestrial ecosystem of China [14], which was in accordance with our results. It is reasonable to use this feature as an indicator for finding the balance between N and P because N: P stoichiometry indicates the nutrient balance from species to ecosystem level and is correlated to vegetation functioning and the physiological traits of plants [40]. The N level was lower for Gramineae, and the P level was lower for Compositae. Our results also demonstrated that there were different responses to different plant functional groups after fertilization, and this indicated that the effect of fertilization on resource allocation strategies was different among species [41].

Our results found that the combination of N and P could enhance the recovery of degraded grassland. However, the interaction between N and P was not significant. This results was consistent with the results of other studies in Alpine meadow [30]. It could because N or P element affects the absorbation and superession in the Tibetan Plateau.

There was nonsignificant influence on biomass for moisture, this tendency is not consistent with the findings of an ecosystem above 3750 m[42] and other ecosystems [42,43], which found that moisture had positively correlated with biomass. In our experiment site, the nutrients may be the essential limited factors for steppe recover. And our results showed that moisture had positively correlated with SOM. With the increasing of SOM, the soil structure will be ameliorated, which improve the soil porosity; on the other hand, the soil colloidal state can be changed to enhance the soil sorption ability [44,45]. The imporvement of SOM and moisture all could increase the available of K in our experiment. The process of K^+^ release is initiated by a low K^+^ concentration in the soil solution and not by cation exchange [46]. With increasing of release K+, plant could absorb more kalium. And kalium enhances the uptake of water by the roots and the water economy of the plant in general. This could explain the reason of the nonignificant influence of soil moisture on the biomas.

Our results showed there was negatively correlation between AP, NH_4_^+^-N, soil moisture with pH, which indicated the application of fertilization initially acidified the soil. This results showed that application of N or P fertilizer alonel decrease the quality and quantity of our experiment site. It is well known that N fertilisers acidify soils [47], while the addition rate that acidify the soil was different in different ecosystems [48,49].

To ameliorate the quality of steppe where Gramineae is the dominate species, 7.5 g N m^-2^ yr^-1^ and 15 g P_2_O_5_ m^-2^ yr^-1^ may be the optimal chioce, since higher addition rate could decrease the ratio of Gramineae to Compositae. While 15 g N m^-2^ yr^-1^ additon was effective where Compositae is the dominate species. And any application rate of P could decrease the quality of steppe in site II. Compared to other communities, Gramineae had a more sensitive response and greater relative dominance because it has a higher nutrient use efficiency when there are sufficient nutrients, but a lower nutrient use efficiency when there are fewer nutrients available [50]. Our results was in accordance with this hypothesis, and also replenish that the addition of P could decrease the relative dominance of Gramineae if it was on inferior position.

## Conclusion

Our study showed that N&P addition had all ameliorated the quality of the two sites in the Tibetan Plateau steppe. N addition led to a large increment in Gramineae and total biomass in the two sites. The addition of phosphorus fertiliser had different influence on the two sites due to the site-species. We did not find strong evidence for critical threshold within 15 g.N m^-2^ yr^-1^, and there was decreasing tendency when P addition rate was above 15 g m^-2^ yr^-1^. The interaction between N and P was nonsignificant for most functional groups.
